# Evolution of the Transforaminal Lumbar Interbody Fusion (TLIF): From Open to Percutaneous to Patient-Specific

**DOI:** 10.3390/jcm13082271

**Published:** 2024-04-14

**Authors:** Peter N. Drossopoulos, Favour C. Ononogbu-uche, Troy Q. Tabarestani, Chuan-Ching Huang, Mounica Paturu, Anas Bardeesi, Wilson Z. Ray, Christopher I. Shaffrey, C. Rory Goodwin, Melissa Erickson, John H. Chi, Muhammad M. Abd-El-Barr

**Affiliations:** 1 Division of Spine, Department of Neurosurgery, Duke University, Durham, NC 27710, USAtroy.tabarestani@duke.edu (T.Q.T.); muhammad.abd.el.barr@duke.edu (M.M.A.-E.-B.); 2 Department of Neurological Surgery, Washington University, St Louis, MO 63110, USA; 3 Division of Spine, Department of Orthopedic Surgery, Duke University Medical Center, Durham, NC 27710, USA; 4 Department of Neurosurgery, Brigham and Women’s Hospital, Boston, MA 02115, USA

**Keywords:** MIS, PLIF, TLIF, transfacet, Kambin’s Triangle, neurosegmentation

## Abstract

The transforaminal lumbar interbody fusion (TLIF) has seen significant evolution since its early inception, reflecting advancements in surgical techniques, patient safety, and outcomes. Originally described as an improvement over the posterior lumbar interbody fusion (PLIF), the TLIF began as an open surgical procedure, that notably reduced the need for the extensive neural retractation that hindered the PLIF. In line with the broader practice of surgery, trending toward minimally invasive access, the TLIF was followed by the development of the minimally invasive TLIF (MIS-TLIF), a technique that further decreased tissue trauma and postoperative complications. Subsequent advancements, including Trans-Kambin’s Triangle TLIF (percLIF) and transfacet LIF, have continued to refine surgical access, minimize surgical footprint, and reduce the risk of injury to the patient. The latest evolution, as we will describe it, the patient-specific TLIF, is a culmination of the aforementioned adaptations and incorporates advanced imaging and segmentation technologies into perioperative planning, allowing surgeons to tailor approaches based on individual patient anatomy and pathology. These developments signify a shift towards more precise methods in spine surgery. The ongoing evolution of the TLIF technique illustrates the dynamic nature of surgery and emphasizes the need for continued adaptation and refinement.

## 1. Introduction

The evolution of the transforaminal lumbar interbody fusion (TLIF) is rooted in the pioneering work of Cloward, Briggs, and Milligan, who introduced the posterior lumbar interbody fusion in 1943 (PLIF) [1,2]. Despite its early promise, the PLIF had limitations, namely a high complication rate and challenging recovery [3]. Harms and Rolinger’s introduction of the TLIF in 1982 marked a pivotal moment in the history of lumbar fusion surgery and emerged as a direct response to the shortcomings of the PLIF, offering a less traumatic and safer unilateral approach to interbody fusion. Its inception was driven by the critical need to enhance patient safety, reduce surgical complications, and improve outcomes in the treatment of lumbar segmental instability and degenerative spinal conditions [4]. The TLIF’s unique method of addressing the shortcomings of the PLIF led to its rapid acceptance and continuous refinement within the surgical spine community. Two key innovators, Leon Wiltse and Parviz Kambin, would later go on to describe two other novel surgical approaches enhancing neural element avoidance by accessing the spinal column in a more lateral fashion—unbeknownst to these pioneers, these key advancements would lay the groundwork for the future of minimally invasive techniques [5,6]. In this narrative review, we will discuss the evolution of the TLIF, tracing its journey from open techniques to minimally invasive and percutaneous techniques. We will also introduce the concept of ‘patient-specific’ TLIF, tailoring this surgical approach in accordance with patient symptomology and high-definition assessment to optimize posterior lumbar interbody fusion.

## 2. Posterior Lumbar Interbody Fusion

The PLIF, as it was first described, inherent to the midline access trajectory, required spinous process and interspinous ligament resection, bilateral laminectomy, and extended neural element (thecal sac and bilateral traversing nerve roots) retraction. Following this extensive decompression, the area between the pedicles of contiguous segments is exposed and the underlying disc space may be accessed with neural retraction. Due to extensive osteotomy and complete discectomy, two cages are often seated into the prepared disc space for the maintenance of early postoperative stability.

### Limitations

Although this technique allows for excellent visualization of target anatomy and circumferential decompression without necessitating patient repositioning, the PLIF was not without major limitations and disadvantages. Due to the aforementioned extensive bony work and neuromuscular retraction, the PLIF is associated with increased operating room time, increased inadvertent durotomy incidence, and increased nerve root injury, when compared to its more recent and lateral adaptations for lumbar fusion [3,7].

## 3. Open Transforaminal Lumbar Interbody Fusion

Transforaminal lumbar interbody fusion soon emerged as a stepwise advancement of the PLIF, directly addressing many of its major limitations and improving clinical outcomes. Popularized by Harms and Rolinger, the TLIF was a unilateral means of achieving the same degree of circumferential fusion as the PLIF technique [4]. Owing to the unilateral nature of this advancement, the TLIF, through a paramedian skin incision, achieved its namesake transforaminal access without the extensive neural retraction required by its predecessor. With this transforaminal approach, the TLIF procedure typically involves removing a portion of the lamina and facet to access the disc space, followed by the removal of the (often damaged) disc and replacing it with bone graft and an Interbody to facilitate fusion and provide postoperative stability. In comparison to its predecessor, the TLIF notably requires only unilateral laminectomy and inferior facetectomy, thereby preserving these structures contralaterally. Taken together, the advantages with this more lateral access trajectory are clear—(1) nerve retraction is limited to only one side, thereby decreasing the risk for iatrogenic injury; (2) the contralateral unperturbed bony structures act as additional surfaces for the fusion construct; and (3) bilateral decompression may be achieved without inheriting the associated morbidities of additional soft tissue, muscle, and neural manipulation on the contralateral side [8]. Later, nerve retraction will, once again, serve as a key differentiator of a future TLIF adaptation [9]. Key to, and coinciding with, advancements in surgical materials including bone grafts and interbody cages, the TLIF was a less traumatic means for correcting the same breadth of spinal pathology as the PLIF [10]. The improvements offered by the TLIF, compared to the PLIF technique, were intuitive.

### Limitations

**Figure 1:**
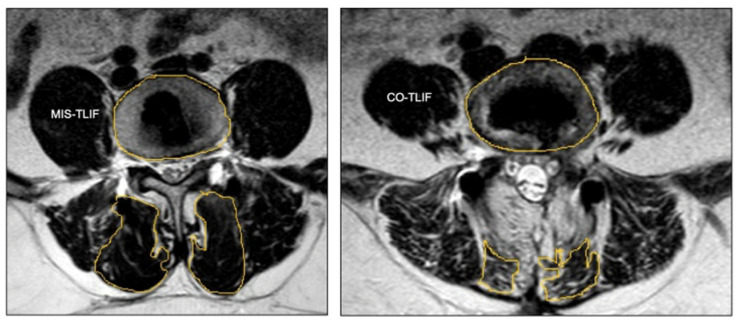
Representative images demonstrating statistically significant postoperative reductions in multifidus (encircled at the bottom of each panel) lean muscle mass in MIS-TLIF versus conventional open TLIF (CO-TLIF). Reproduced in its original form from Global Spine Journal, Volume 14, Dave et al. [13], Does Conventional Open TLIF cause more Muscle Injury when Compared to Minimally Invasive TLIF?—A Prospective Single Center Analysis, 93–100, Creative Commons Copyright 4.0 (2024), Sage Journals.

The TLIF quickly rose to become a cornerstone procedure in treating degenerative disease. However, it was still plagued by paraspinal iatrogenic injury, due to the extensive period of muscle retraction, which often results in long recovery periods, albeit to a lesser degree than the PLIF [11,12]. In fact, many previous studies comparing the well described, long-term postoperative paraspinal muscle degeneration associated with the traditional open TLIF found that its minimally invasive counterpart demonstrated clear superiority in this avenue (Figure 1) [13,14,15,16].

## 4. Minimally Invasive Transforaminal Lumbar Interbody Fusion

**Figure 2:**
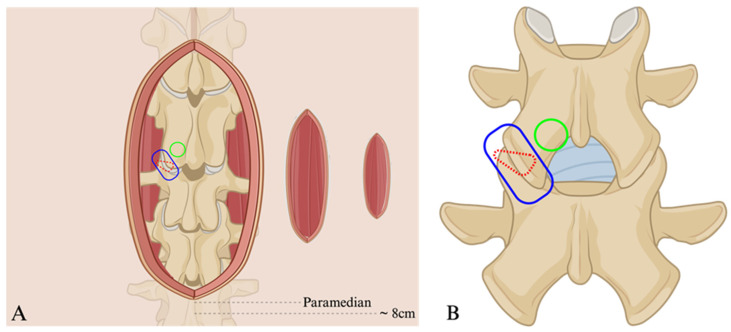
Animated depiction of various access trajectories at the level of the skin (**A**) and target vertebral level (**B**). Wide midline incision (**A**) representing the exposure for the PLIF and open TLIF. More laterally, paramedian is a representation of the skin incision for the MIS-TLIF and the transfacet LIF; most laterally is the representative incision for the Trans-Kambin’s Triangle TLIF at approximately 8 cm from midline. The green circle highlights (**A**,**B**) the point of access osteotomy for the TLIF and blue encircles the facet joint, which represents the transfacet corridor. Finally, the red dashed triangle is a depiction of where Kambin’s Triangle would lie from a lateral viewpoint—dashes indicate that the triangle is not viewable from this posterior angle. Figure created using BioRender.com.

In the pursuit of less traumatic, but equally effective, means of lumbar stabilization, Harms and Rolinger’s initial TLIF technique faced growing concerns due to exposure-related adverse events [17]. Although improved upon from the PLIF, the TLIF still requires extensive retraction and muscle stripping, which leads to soft tissue injury, prolonged postoperative pain, and suboptimal outcomes [18,19]. Recognizing the need for change and born out of innovations like the tubular retractor, Kevin Foley and Michael Lefkowitz introduced the concept of minimally invasive TLIF (MIS-TLIF) in 2002 [20]. This adaptation revolutionized the posterior approach to lumbar interbody fusion by minimizing soft tissue damage. Foley and Lefkowitz’s MIS-TLIF introduced two mirroring 1 inch paramedian incisions (Figure 2), enabling bilateral facet joint access, discectomy, interbody implant placement, and bone grafting through a tubular retractor. Subsequent percutaneous fixation through these incisions further reduced soft tissue disruption and refined the traditional TLIF to a technique with a reduced surgical footprint [17]. This innovation marked a significant shift towards minimally invasive approaches in spine surgery [17].

The adaptation of minimally invasive techniques for lumbar interbody fusion ushered in an era of technological advancements, namely in interbody design and advanced imaging. Expandable interbody cages are engineered for insertion with a small profile and then expanded within the evacuated disc space. This design aims to achieve a better fit between vertebral endplates, addressing the issues and complications often associated with static cages [21,22]. Further, these expandable cages allow for indirect contralateral decompression, which obviates the need for direct bilateral decompression [23]. The smaller size of these devices when contracted translates to less insertional force, thereby maintaining the integrity of the vertebral endplates. Further, the insertion of these devices requires less nerve root retraction and potentially reduces postoperative complications such as cage subsidence [21]. Similarly, the MIS-TLIF platform has become a cornerstone for integrating cutting-edge imaging technologies such as robotic assistance, 3D neurosegmentation, and augmented reality [24,25,26]. These advancements have solidified the role of the MIS-TLIF as a fundamental framework for ongoing research and development in spine surgery. Further, this progression sets the stage for exploring advanced operative techniques, such as those embodied in the Trans-Kambin’s Triangle TLIF (percLIF), furthering the pursuit of less destructive, yet effective, interbody fusion methods.

### 4.1. Outcomes

Perhaps most importantly, we must evaluate the outcomes of such adaptations to the TLIF and ensure that their less immediately destructive nature does not incur the expense of a poor safety profile or worse outcomes. Although the MIS access trajectory is identical to that of the open TLIF, the narrow-angle view of target anatomy through tubular retractors makes this technique technically challenging. However challenging, the MIS-TLIF offered the potential to improve clinical outcomes without compromising patient safety.

In the largest cohort study comparing minimally invasive to open TLIF in the treatment of grade 1 degenerative lumbar spondylolisthesis, Chan et al. found that the MIS-TLIF resulted in a lower estimated blood loss (108.8 mL versus 299.6 mL, *p* < 0.001) and longer operative times (228.2 min versus 189.6 min, *p* < 0.001) [27]. Additionally, fusion success, as well as 30 day and 5 year complication rates (including reoperation and instrument revision, subsidence, pseudoarthrosis, and adjacent segment disease) were comparable among these cohorts [27]. Although the MIS cohort exhibited lower Oswestry Disability Index (ODI) and pain scores (VAS) than their open TLIF counterparts, multivariable analysis indicated no significant differences in these long-term outcomes [27]. This large multicenter study echoes the outcomes of other studies with a shorter average follow-up [28,29,30,31,32,33]. With respect to postoperative complications and patient reported outcomes, it is clear that the MIS-TLIF is at least as safe and effective as the traditional open technique.

Less consistent in the literature, however, are reports on length of stay and operative time. With respect to length of stay, many studies, including the previously cited Chan et al. cohort study, report no significant length of stay differences while others found that the MIS technique is associated with shorter lengths of stay—perhaps protocolized institutional variations in postoperative care for these patients exist that may explain length of stay variations [34]. The trends for operative duration are similarly variable—a large meta-analysis of over 2300 patients found no statistically significant differences between open and minimally invasive TLIF surgery (198 min versus 214 min, respectively) [27,32,33,34,35]. Many of the published studies comparing open to MIS TLIF outcomes are not single surgeon studies and do not comment on the comfort or proficiency of the surgeons who contributed operative cases. Taken together with the demonstrated learning curve associated with the MIS TLIF, it is reasonable to suggest that operative time variability may be confounded by position on the MIS learning curve and surgeon-to-surgeon variability [27]. Future studies should aim to control such confounders to better characterize these techniques.

Finally, we must compare the objective spinopelvic parameter improvements of these techniques to explore whether or not the stepwise TLIF advancements conferred improvement, or at least maintenance, of radiographic parameters. In a recent radiographic study of 267 propensity score-matched patients (114, 43% MIS-TLIF), Dibble et al. demonstrated that, compared to the open TLIF cohort, MIS-TLIF recipients experienced significantly larger improvements in sacral slope change (4.14 ± 4.35 degrees vs. 1.15 ± 3.88 degrees, *p* < 0.001) and anterior disc height change (4.25 ± 3.68 mm vs. 1.41 ± 3.77 mm, *p* < 0.001) [36]. MIS-achieved lumbar lordosis additionally improved over open TLIF, although this trend was not statistically significant [36]. Other, smaller studies share similar findings, MIS-TLIF is at least as ‘corrective’ as open TLIF [37,38].

### 4.2. Limitations

The MIS-TLIF continues to serve as a robust platform for innovation in spine surgery; however, there are notable limitations which warrant discussion. As previously mentioned, and inherent to the nature of minimally invasive surgery, the associated decreased surgical footprint is afforded at the expense of decreased direct anatomic visualization and surgical field of view at the target operative sites—the principal limitation of the MIS-TLIF. To this effect, the MIS-TLIF is technology dependent—it is only as powerful as the ‘indirect’ visualization provided by the imaging modality facilitating surgery. Accordingly, imaging systems, namely traditional fluoroscopy and computed tomography (CT) navigation, are employed and facilitate safe visualization of target anatomy. Unfortunately, these modalities introduce another notable limitation—radiation. In many cases, the radiation burden to the patient and operative team may be significant. In fact, through a large meta-analysis investigating MIS-TLIF and open TLIF radiation exposure, Kim et al. revealed a 2.4-fold increased radiation burden to the patient in the MIS cohort [39]. The radiation burden to the operative team is similarly notable—several studies have even correlated surgical subspecialties exposed to high cumulative radiation burdens to increased incidence of cancer, compared to the general population [40]. Though significant, warranting further investigation, there are novel instrument tracking technologies emerging, which aim to minimize radiation exposure without compromising visualization.

Finally, it is important to introduce the concept of ‘hidden’ blood loss (HBL); that is, blood loss not accounted for by intraoperative estimates or postoperative drain volumes. Although HBL was first described in 2000 in knee arthroplasty patients, only recently has this entity been investigated in spine surgery [41]. This is particularly germane to MIS spine surgery, where surgeons do not always elect to place postoperative drains. In many cases, hidden blood loss may be substantial; several studies have reported that HBL for MIS-TLIF accounted for near 50% of the total blood loss for a patient [42,43,44]. However, when comparing the HBL and total blood loss (TBL) of MIS-TLIF patients to that of open TLIF patients, Yang et al. found that although open TLIF results in a greater magnitude of HBL and TBL than MIS-TLIF, the ratio of HBL to TBL in both cohorts was similarly approximated, at just under 50% (*p* = 0.626) [43]. Recognizing the deleterious impact of increasing TBL, early identification and management of HBL can preempt clinical deterioration. Thus, awareness and proactive intervention when indicated are essential responsibilities for spine surgeons.

## 5. Trans-Kambin’s Triangle Lumbar Interbody Fusion

Building upon the principles of minimal invasiveness and precision in lumbar stabilization, the percLIF approach, rooted in the utilization of Kambin’s Triangle, offers another perspective in the ongoing evolution of spine access. Originating from Parviz Kambin’s work in the early 1980s, percLIF, as a means of lumbar interbody fusion, has been shaped by both historical insights and modern technological advancements, presenting unique advantages and challenges in pursuit of optimal fusion solutions [6]. In this early description, beginning some 10 cm off midline, Kambin’s Triangle was used as the path for an arthroscope to alleviate nerve root compression by aspirating herniated disc material [6]. Later that decade, Kambin would go on to define the borders of the anatomical triangle that would bear his name—the exiting nerve root, the superior articular process of the inferior vertebrae, and the superior end plate of the inferior vertebral body [45]. This technique was soon adapted as a means for foraminoplasty, decompression of spinal canal stenosis, and, ultimately, for interbody fusion [46]. Today, largely facilitated by interbody devices and imaging technology advancements, it is common to find surgical approaches for interbody fusion described through Kambin’s Triangle. It is important to note, however, that the variation in the size of this anatomic triangle, as shown by anatomical studies, has raised concerns about the safety of blindly placing large interbody cages through a relatively small corridor [24].

Again, advancements in 3D imaging technology are transforming spinal surgery, offering high-resolution surgical planning; this is especially pertinent in the context of Kambin’s Triangle TLIF. However, despite these technological strides, anatomical studies reveal limitations in the practical application of Kambin’s Triangle for interbody fusion. For instance, in a large anatomic study, only 4% of measured triangles could accommodate the appropriate cannula sizes required for interbody placement, contraindicating this approach [47]. This has led to the exploration of alternative methods for cases requiring larger cages or where traditional access paths are less feasible. While this technique must be selectively applied in the appropriate clinical and anatomic context, it is nonetheless a safe and efficacious means of interbody fusion, becoming an integral member in the array of fusion strategies [48,49,50,51]. These advancements in imaging and surgical planning, while promising, underscore the ongoing need to adapt and refine surgical techniques in response to variable anatomic patient constraints.

**Figure 3:**
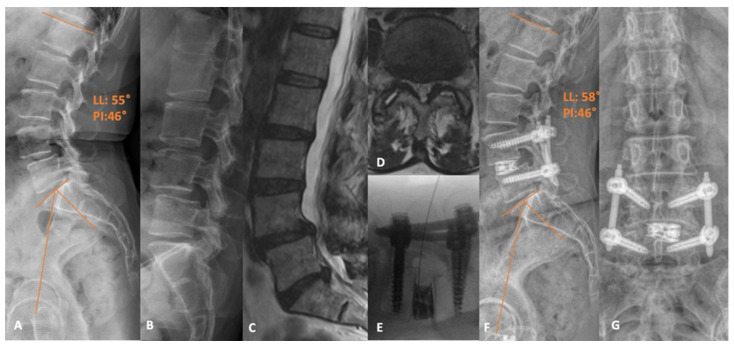
(**A**) Preoperative standing X-ray demonstrating the L4-5 grade 1 spondylolisthesis, with (**B**) flexion films demonstrating dynamic instability. (**C**) Sagittal and (**D**) axial T2-weighted MRI slices revealing the advanced central and bilateral recess stenosis at the L4-5 level. (**E**) Intraoperative fluoroscopic image following cage placement with a guidewire within the disc space. (**F**) Postoperative lateral and (**G**) AP X-rays supporting a satisfactory appearance of the construct. LL: Lumbar lordosis. PI: Pelvic incidence.

Below, the authors share their institutional experience and present a representative percutaneous Trans-Kambin’s Triangle TLIF case. Case 1 is a 67-year-old female with a long-standing history of low back pain radiating down both lower extremities. Preoperative imaging demonstrated a significant mobile spondylolisthesis at L4-5 and an accompanying severe central and bilateral recess stenosis (Figure 3). Conservative treatment with physical therapy and injections failed to provide adequate relief and she elected to proceed with surgery. We ultimately offered and proceeded with a percutaneous L4-5 trans-Kambin’s Triangle interbody fusion. The surgery was uncomplicated and completed in just over 2 h. She was ultimately discharged home later that day and reported significant symptomatic improvement at her most recent follow-up visit (1 year).

### 5.1. Percutaneous Endoscopic Transforaminal Lumbar Interbody Fusion

To address the lack of direct intraoperative visualization with the Trans-Kambin’s Triangle TLIF, Nagahama et al. proposed a modified procedure, deploying the endoscope (rather than relying solely on imaging for guidance) [52]. Their procedure was coined percutaneous endoscopic transforaminal lumbar interbody fusion (PETLIF). By employing an endoscope, the authors were able to directly visualize entrance into the disc space through Kambin’s Triangle and avoid major vascular or neurologic injury. Further, the authors highlighted that, at the time of publication, this was the only procedure that could safely provide indirect decompression using an oval dilator (versus expandable cage systems) [52].

### 5.2. Outcomes

Since the inception of percutaneous means for access to the disc space through Kambin’s Triangle, multiple studies have compared the relevant clinical and surgical outcomes of this technique to its longer standing predecessor, the MIS-TLIF [53]. In a prospective cohort study of 75 patients, Ao et al. found that true intraoperative blood loss, measured plus hidden, and the postoperative visual analog scale for back pain were significantly improved in the PETLIF cohort relative to their MIS-TLIF counterparts (*p* < 0.001) [53]. Similarly, in a later study of 16 patients who underwent percLIF for grade 1 spondylolisthesis correction, Wang et al. found that all patients maintained anterior and posterior disc height correction, spondylolisthesis correction, and remained complication-free (including permanent neurological injury, infection, and deep venous thrombosis) at their 12 month follow-up [54]. Moreover, Wang et al. reported significant improvement over preoperative ODI and successful fusion at 1 year for 15/16 patients [54]. Finally, in a larger review considering 17 studies, Ono et al. echoed these findings—the percutaneous TLIF technique minimizes the surgical footprint, reduces postoperative pain, reduces blood loss, and is associated with faster recovery times compared to the MIS-TLIF [55].

### 5.3. Limitations

Echoing the imitations inherent to MIS spine surgery described in Section 4.2, percutaneous approaches to the disc space through Kambin’s Triangle are constrained by the balance of decreased direct visualization and radiation exposure. Specific to endoscopic approaches, however, there is a notable learning curve [56,57]. As expected, operative time significantly improves as a surgeon becomes more facile with the endoscope [57]. Importantly, surgical outcomes including failure and disease recurrence remain stable along the learning curve [56,57].

## 6. Transfacet Lumbar Interbody Fusion

The transfacet LIF (TF-LIF) was first described in 2020 by Khalifeh et al. as a modification to the MIS-TLIF [58]. Uniquely, during TF-LIF, the intervertebral disc space is accessed entirely through the bony corridor provided by the flanking facets, which nearly eliminates the feared percLIF complication of nerve root violation. While maintaining the medial inferior articular process, lateral superior articular process, and rostral pars interarticularis, limited bony resection of the superior articular process provides access to the disc space without exposing the traversing nerve root. Using the transfacet trajectory, surgeons are equipped to supersede the space limitations of the percLIF through facet resection. Like percLIF though, TF-LIF also allows for direct visualization of the endplate while preparing the disc space for an implant [59]. Again, it is important to state that the transfacet approach to the disc space carries the same limitations applicable to MIS-TLIF and percutaneous approaches to the disc space through Kambin’s Triangle, as described in Section 4.2 and Section 5.3. Additionally, this technique is constrained by the unique facet anatomy of the individual patient, which must be large enough to safely pass an interbody and prepare the endplates.

**Figure 4:**
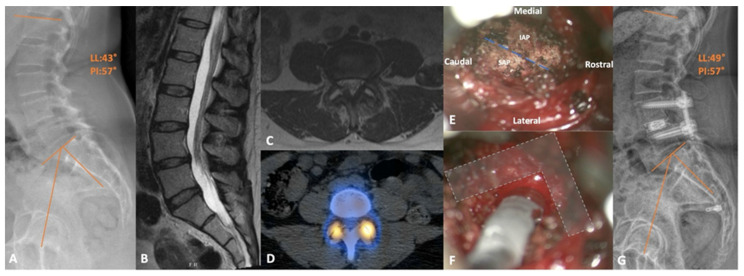
(**A**) Preoperative standing lateral X-ray demonstrating the L4-5 grade 1 spondylolisthesis. (**B**) Sagittal and (**C**) axial T2-weighted MRI showing the mild central and severe lateral recess stenosis, respectively, with the latter image depicting the bilateral facet joint effusion. (**D**) Axial slice of the CT SPECT scan showing the isolated increase in radiotracer uptake at both L4-5 facet joints. (**E**) Intraoperative view of a right-sided transfacet approach with the joint line (dashed line), inferior articular process (IAP), and superior articular process (SAP) illustrated. (**F**) The same approach after completing the required facetectomy and discectomy to allow adequate room for cage trials; bony boundaries protecting the neural structures (L shape). (**G**) Postoperative lateral X-ray with adequate reduction in the slip and improved lordosis.

Below, the authors share their institutional experience and present a representative transfacet LIF case. Case 2 is a 55-year-old female, who presented from an outside institution, complaining of lower back and bilateral lower extremity pain. Although her physical exam was unremarkable, preoperative imaging was significant for a mobile spondylolisthesis at L4-5, accompanied by a facet cyst, mild central canal stenosis, and severe bilateral recess stenosis (Figure 4). After exhausting conservative means of relief, she ultimately elected to proceed with definitive surgical correction. After consideration, we offered and proceeded with a right transfacet lumbar interbody fusion. The procedure was completed without complication in under 3 h. She was ultimately discharged home on the first postoperative day and reported significant pain subsidence at her most recent follow-up visit (6 months).

### Outcomes

Though the TF-LIF is an emerging technique, and its novelty precludes long-term investigations, the early reports on its safety and outcomes are consistent and robust, clearly demonstrating restoration of spinopelvic parameters and improvement in patient-reported outcomes [60,61]. In the largest study of TF-LIF patients, Khalifeh et al. reported commendable 12-month outcomes for their cohort of 68 patients [60]. Specifically, they found that the TF-LIF resulted in significantly improved ODI and VAS back pain scores [60]. Further, Khalifeh et al. reported improvements in disc height (0.71 cm), segmental lordosis (6.83 degrees, 9.10 degrees in patients with severe preoperative hypolordosis), and lumbar lordosis (8.65 degrees) [60]. With respect to complications, subsidence was observed in 6/74 (8.1%) levels and 94.3% patients demonstrated radiographic evidence of fusion by 12 months [60]. To further characterize the TF-LIF, further investigation via prospective study and comparison to traditional techniques is required.

## 7. Patient-Specific Lumbar Interbody Fusion

### 7.1. Introduction

As evidenced in the discussion above, the evolution of these TLIF adaptations over the years represents significant advancement for patients and surgeons alike. In the earlier years of lumbar fusion, surgical options were limited, which, in hindsight, may not have been best suited for a specific patient’s pathology and anatomic parameters. The diversification of TLIF methods has brought increased responsibility upon the surgeon to accurately select the approach that will balance surgical footprint and patient safety with the highest chance of achieving symptomatic improvement [62]. In this light, novel advanced imaging technologies have been developed, allowing surgeons to preoperatively segment each of the aforementioned corridors to better assess the risks and benefits of each technique in a patient-specific fashion [24]. In the broader context of spine surgery, the integration of such a technology into the operating room is somewhat delayed compared to other subspecialities in and out of neurosurgery. For example, functional, skull base, and intracranial operations have relied heavily on the preoperative segmentation of cortical tracts, thalamic nuclei, and tumor boundaries to minimize complications [63,64,65]. Afforded by the advent and advancement of these imaging modalities, minimally invasive approaches in the appropriate clinical context are increasingly employed.

### 7.2. Three-Dimensional Neurosegmentation

The initial implementation of spine segmentation technology was primarily aimed at helping surgeons avoid nerve roots during percutaneous TLIF [25]. However, its application has since expanded to include both anatomic studies of Kambin’s Triangle and delineating borders for the TF-LIF. More importantly, this technology has recently been implemented to facilitate selecting the ‘ideal’ access trajectory, dictated by a patient’s unique anatomy. ‘Ideal’, in this sense, references the following: (1) the measured maximum allotted cannula diameter to introduce the desired interbody cage with appropriate dimensions; (2) calculating the area of each corridor, considering the bordering neurovascular structures; (3) identifying potential anatomic anomalies like conjoined nerve roots; and (4) comparing the areas of each safe corridor to reduce the risk of iatrogenic injury. Tabarestani et al. showcased the first practical application of this technology in a small case series where Kambin’s Triangle volumes were measured for all patients bilaterally at the level of operation [26]. By comparing contralateral areas, the surgeon was able to decide on the ‘ideal’ laterality of their approach, entering the largest, safest triangle with the greatest distance from the exiting nerve root. Further applying this same methodology, the same research group examined how various pathologies affect Kambin’s Triangle; they discovered that higher levels of spondylolisthesis, with decreased posterior disc space heights, minimize the safe area of entry for the percLIF [25]. Further work is required to similarly describe how certain pathology might favorably augment other access planes.

**Figure 5:**
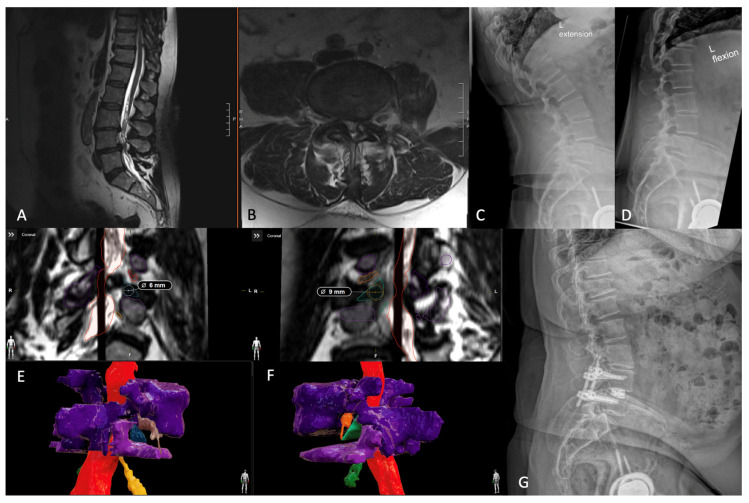
(**A**,**B**) Preoperative T2-SPACE-weighted 1 mm slice MRI showing L4-5 grade 1 spondylolisthesis on both sagittal and axial views. (**C**,**D**) Preoperative extension and flexion radiographs further depicting the instability of the pathologic L4-5 facet joints. (**E**) BrainLab’s planning software highlighting the left trans-Kambin corridor (outlined in blue) and (**F**) the right trans-Kambin corridor (outlined in green), with the maximum permissible cannula measurement overlaying each area and 3D reconstrued images below. (**G**) Postoperative radiograph confirming appropriate hardware placement and correction of their spondylolisthesis.

Below, the authors present a case highlighting the use of this patient-specific technology to identify the safest laterality into the disc space via Kambin’s Triangle. Case 3 is a 65-year-old male with grade 1 spondylolisthesis at L4-5, who previously failed conservative measures for his back pain and bilateral leg radiculopathy. Preoperative MRI imaging revealed cephalad migration of the extruded disc at L4-5 with severe central canal stenosis and disc protrusion, causing severe right neuroforaminal narrowing. Given the fact that previous facet injections provided significant but transient relief, he was offered an awake TLIF under spinal anesthesia. Due to his bilateral facet arthropathy, the team decided to segment the patient’s spine to obtain better visualization of both Kambin’s Triangles to access the disc space safely. The segmentation was carried out using high-resolution T2 MRI-SPACE sequences in BrainLab (Munich, Germany), as has been previously described in the literature [26]. Based on measurements of both the maximum permissible cannula diameter and safe area, the right percLIF approach was chosen, given that it comprised the largest dimensions (9 mm and 101 mm^2^, respectively). The surgery was completed with 50 mL estimated blood loss, without complication, and the patient was safely discharged home on postoperative day 1. The postoperative course was largely uneventful; his radiculopathy and spinopelvic parameters improved significantly (Figure 5).

Furthermore, segmentation technology has found applications beyond the percLIF approach. Initially described by Khalifeh et al., the TF-LIF generally provides a larger working area for disc space access than Kambin’s Triangle and facilitates the implantation of a larger interbody device [58,60]. In a follow-up study, Tabarestani et al. used segmentation to assess and compare the safe triangles of the traditional open TLIF and MIS-TLIF, Kambin’s Triangle, and Trans-facet corridors for 11 patients [24]. In this proof-of-concept study, their findings revealed not only increased permissible cannula diameters and mean areas advantageous for the TF-LIF approach, but also illustrated the ability to preoperatively delineate key anatomy, particularly nerve roots, informing operative decision making with special attention to avoiding feared iatrogenic injuries at these levels. While this technology marks a significant advancement, there remains room for improvement before these techniques can be implemented on a larger scale. Namely, the automation of the segmentation process is necessary to reduce manual error and improve workflow [66]. However, until companies are able to produce accurate 3D segmentations of patient spines with overlaying neurovascular structures, manual segmentation on a patient-by-patient basis will remain a valuable tool for surgeons to facilitate patient selection and surgical planning.

**Figure 6:**
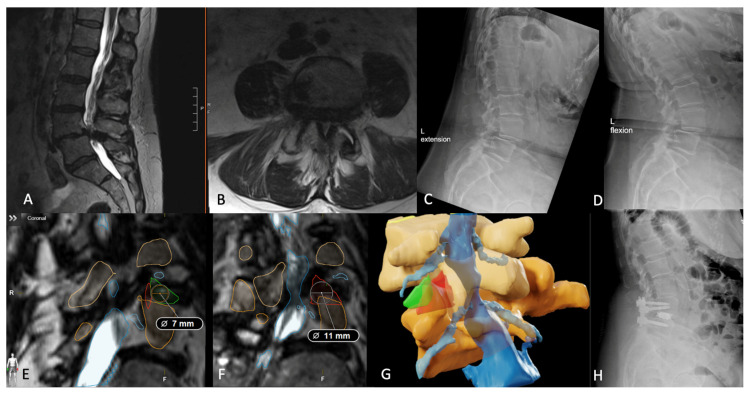
(**A**,**B**) Preoperative T2-SPACE-weighted 1 mm slice MRI showing L4-5 grade 1 spondylolisthesis on both sagittal and axial views. (**C**,**D**) Preoperative extension and flexion radiographs, further depicting the instability of the pathologic L4-5 facet joints. (**E**) BrainLab’s planning software highlighting the left trans-Kambin corridor (outlined in green) and (**F**) the left trans-facet corridor (outlined in red) with the maximum permissible cannula measurement overlaying each area. (**G**) 3D reconstructed image based on the segmented objects displaying the overlapping left TF-LIF (green) and percLIF (red) approaches. (**H**) Postoperative radiographs confirming appropriate hardware placement and correction of their grade 1 spondylolisthesis.

Below, the authors present a case highlighting the use of this patient-specific technology in determining the ‘ideal’ approach for surgery when the percLIF approach was determined too small for safe entry. Case 4 is a 61-year-old female with grade 1 spondylolisthesis at L4-5 who previously failed conservative measures for her back pain and left leg radiculopathy. Preoperative MRI revealed multilevel degenerative changes of the lumbar spine, worst at L4-5 with a 6 mm anterolisthesis and severe facet arthrosis, causing severe canal stenosis and buckling of the proximal nerve roots. Given that her previous L4-5 facet injections provided temporary relief, she was offered a TLIF to correct her misalignment. Due to the nature of her severe facet arthrosis, with widening of the left facet joint and narrowing of the lateral recesses bilaterally, the team segmented the patient’s spine to better visualize potential corridors for safe disc space access [26]. Based on measurements of both the maximum permissible cannula diameters and safe working areas, the left TF-LIF approach was chosen, given its larger dimensions (10.2 mm and 161.1 mm^2^, respectively) compared to the right TF-LIF trajectory and either percLIF approach. The surgery was completed without complication and 50 mL estimated blood loss. The postoperative course was uneventful; the patient’s radiculopathy improved significantly, spinopelvic correction was attained, and the patient was discharged home 2 days later (Figure 6).

### 7.3. Patient-Specific Implants

Akin to neurosegmentation, descriptions of patient-specific interbody cages are emerging in the literature; however, at the time of writing, such 3D-printed interbody reports are limited to small case studies and complex applications [67,68,69,70,71]. Early cadaveric feasibility studies have demonstrated that patient-specific cages increase contact area and decrease contact stress more so than their ‘off-the-shelf’ counterparts [72]. Although these early reports are promising, a large-scale prospective study is certainly required before such custom cages can become commonplace. Again, in the setting of MIS surgery, direct anatomic visualization by the surgeon is often constricted by the size of their exposure or cannula. Custom cages have the ability to circumvent space restrictions and select more patients for MIS surgery who require larger interbody devices—an interbody device may be designed to achieve the desired spinopelvic parameter correction, while maintaining an MIS-friendly footprint.

Below, the authors present their institutional experience of utilizing patient-specific interbodies through a representative MIS-TLIF case. Case 5 is a 68-year-old male who presents with low back pain radiating to bilateral lower limbs, right greater than left, which severely limits his activity. Further evaluation with flexion–extension films and MRI of the lumbar spine demonstrate that the patient has an L4-5 spondylolisthesis, overgrown facet joints, L4-5 disc height loss, and a facet cyst emanating off the right L4-5 facet joint, causing severe lateral recess stenosis and contributing to central stenosis. At the time of presentation, the patient had not responded to conservative management, including physical therapy, chiropractic management, anti-inflammatories, muscle relaxants, opioids, and epidural steroid injections. Taken together, we ultimately planned for an MIS-TLIF with a custom, 3D-printed, titanium interbody.

The patient received a registration CT scan and lumbar plain films, and a custom-made cage was designed to achieve the maximum anatomically appropriate disc height, improve L4-5 discal lordosis from 2 degrees to 10 degrees and subsequently improve lumbar lordosis to 49 degrees (Carlsmed aprevo, Carlsbad, CA, USA). This titanium interbody was 3D printed and subsequently implanted in standard fashion.

**Figure 7:**
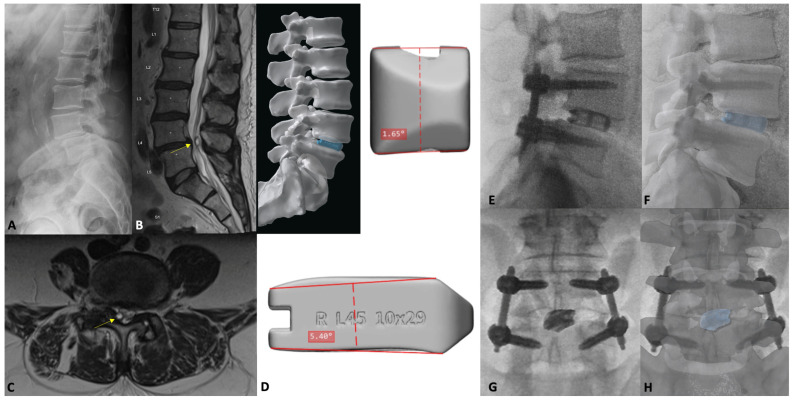
Lateral flexion plain film (**A**) demonstrating the L4-5 spondylolisthesis and disc height loss. Sagittal (**B**) and axial (**C**) T2-weighted MRI depicting the L4-5 facet cyst (yellow arrow) and lateral recess stenosis. Preoperative 3D rendering (**D**) of the patient’s spine and proposed custom L4-5 interbody (Carlsmed aprevo, Carlsbad, CA, USA). Postoperative lateral (**E**) lumbar plain film with overlying custom cage rendering (**F**). Postoperative AP (**G**) plain film with overlying custom cage rendering (**H**).

Postoperatively, this patient’s course was largely uneventful, and he was discharged home on the first postoperative day. L4-5 intradiscal lordosis increased to 12 degrees (from 2 degrees), lumbar lordosis increased to 45 degrees (from 41 degrees), and disc heights improved to 8.5 mm (anterior, from 4.3 mm) and 7.8 mm (posterior, from 3.3 mm). Notably, these parameter improvements were achieved with a 5.4 degree cage (Figure 7).

## 8. Conclusions

The transformative journey of the TLIF from its inception to the cutting-edge adaptations facilitated by technological advancement demonstrates a remarkable evolution in spine surgery, specifically for interbody fusion. Each consecutive iteration of the cornerstone TLIF has progressively enhanced patient safety, reduced recovery time, and improved clinical outcomes. The integration of 3D neurosegmentation technology and advanced imaging into the preoperative workflow has been pivotal and enables surgeons to tailor their technique to the unique anatomical constraints of each patient. This patient-specific evolution represents the pinnacle of precision in spine-surgery and highlights an important shift from the traditional “one-size-fits-all” approach, to more nuanced, tailored treatments. As the field continues to evolve alongside technological innovation, it is clear that the future of spine surgery holds even more refined techniques, reduced complication rates, and improved quality of care for all patients. The ongoing development and improvement of these advanced techniques underscores the dynamic nature of spine surgery and demands continuous learning and refinement by surgeons alike. Thus, the journey of the TLIF mirrors the broader, ever advancing evolution of surgical science.
